# Cryo-EM structure provides insights into the dimer arrangement of the O-linked β-N-acetylglucosamine transferase OGT

**DOI:** 10.1038/s41467-021-26796-6

**Published:** 2021-11-11

**Authors:** Richard W. Meek, James N. Blaza, Jil A. Busmann, Matthew G. Alteen, David J. Vocadlo, Gideon J. Davies

**Affiliations:** 1 York Structural Biology Laboratory, Department of Chemistry, University of York, York, YO10 5DD UK; 2 Department of Molecular Biology and Biochemistry, Simon Fraser University, 8888 University Drive, Burnaby, BC V5A 1S6 Canada; 3 Department of Chemistry, Simon Fraser University, 8888 University Drive, Burnaby, BC V5A 1S6 Canada

**Keywords:** Cryoelectron microscopy, Glycobiology, Transferases

## Abstract

The O-linked β-N-acetylglucosamine modification is a core signalling mechanism, with erroneous patterns leading to cancer and neurodegeneration. Although thousands of proteins are subject to this modification, only a single essential glycosyltransferase catalyses its installation, the O-GlcNAc transferase, OGT. Previous studies have provided truncated structures of OGT through X-ray crystallography, but the full-length protein has never been observed. Here, we report a 5.3 Å cryo-EM model of OGT. We show OGT is a dimer, providing a structural basis for how some X-linked intellectual disability mutations at the interface may contribute to disease. We observe that the catalytic section of OGT abuts a 13.5 tetratricopeptide repeat unit region and find the relative positioning of these sections deviate from the previously proposed, X-ray crystallography-based model. We also note that OGT exhibits considerable heterogeneity in tetratricopeptide repeat units N-terminal to the dimer interface with repercussions for how OGT binds protein ligands and partners.

## Abstract (web summary)

The modification of proteins with O-linked β-N-acetylglucosamine (OGlcNAc) plays roles in regulation of numerous cellular functions while incorrect O-GlcNAcylation patterns are linked to disease. Here, the authors report a cryo-EM structure of full-length O-GlcNAc transferase (OGT), the only enzyme responsible for O-GlcNAcylation.

## Introduction

The modification of hydroxyl groups of serine and threonine residues of proteins with O-linked β-N-acetylglucosamine (O-GlcNAc) is implicated in transcription, translation, signalling, proteasomal degradation and cell homeostasis^1^. Incorrect patterns of O-GlcNAcylation are linked to neurodegeneration and cancers^1–3^. Remarkably, OGT is the sole enzyme modifying thousands of proteins in the nucleus, cytoplasm, and mitochondria^4,5^. The O-linked GlcNAc is not elongated to form longer, complex glycans and instead cycles on and off the polypeptide target, more akin to phosphorylation than classical glycosylation^1^. Indeed, it is suspected crosstalk occurs between phosphorylation and O-GlcNAcylation^1^. Beyond glycosylation, OGT cleaves Host Cell Factor 1 (HCF-1) and functions as an interaction partner in large multi-protein complexes^6–9^.

OGT is composed of an N-terminal region comprising 13.5 tetratricopeptide repeat (TPR) units and a C-terminal multi-domain catalytic region. The catalytic region alone can glycosylate short peptide targets, however, the TPRs are necessary for modification of full-length proteins, promoting peptide cleavage, and engaging in protein-protein interactions^9–11^. Underscoring their importance, detrimental mutations in the TPR region are linked to X-linked intellectual disability (XLID)^2^. XLID is characterised by an inability to understand complex ideas and having limitations in adaptive behaviour, which start before adulthood^12^.

**Fig. 1:**
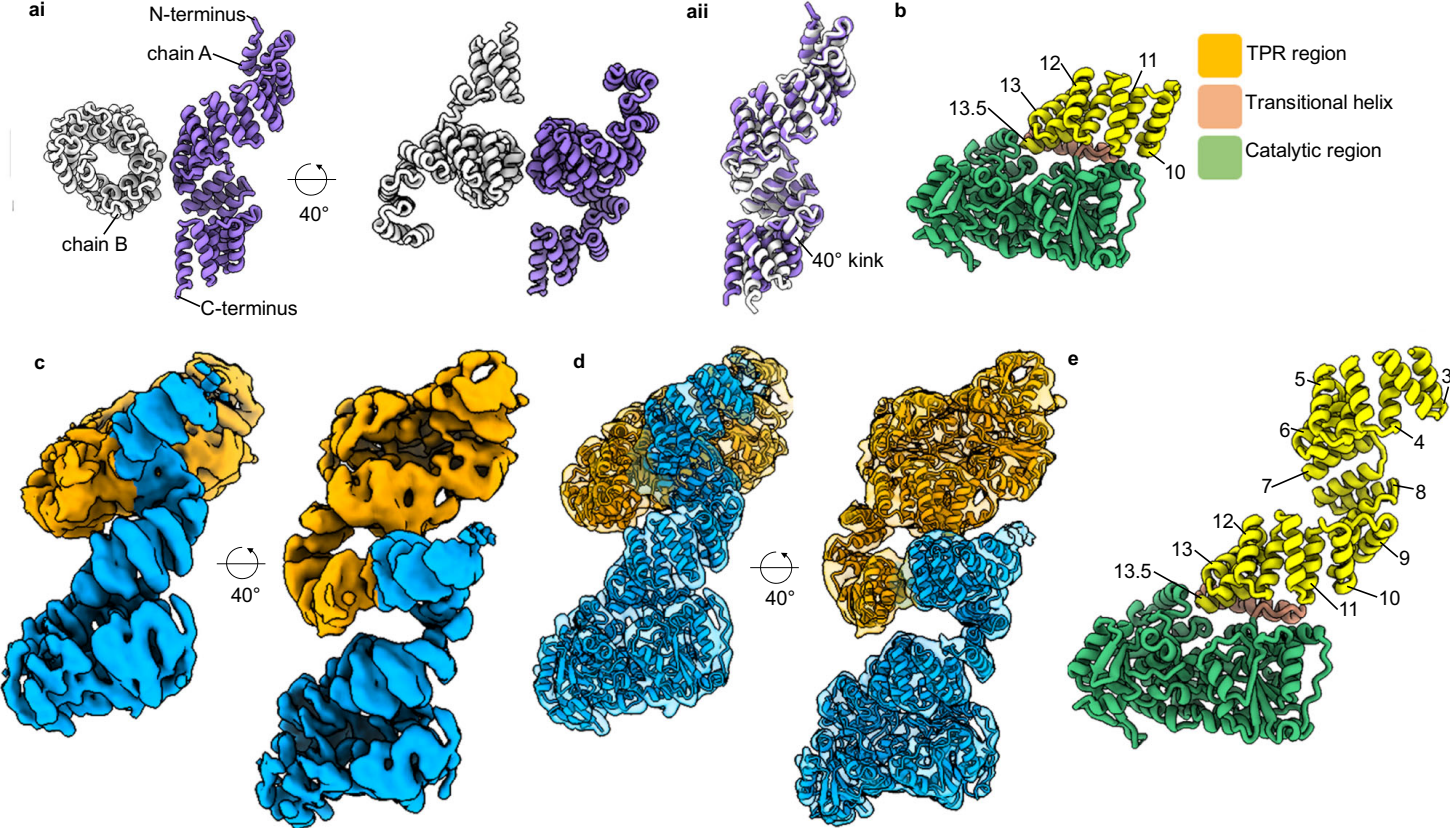
The structure of unliganded OGT. **ai** X-ray structure of the dimeric TPRs 11.5 construct^14^. **aii** Comparison of chain A to chain B demonstrates a 40° kink between chains. **b** X-ray structure of the catalytic domain of OGT appended to 4.5 TPRs, TPRs numbered^13^. **c** 5.3 Å cryo-EM map into which the cryo-EM model of the OGT dimer was fitted (**d**), orange and blue represent separate chains and their associated maps depicted at 40° rotations. **e** Chain A of the dimer assembly and the TPR units observed are numbered.

Understanding the full-length structure of OGT, and how it interacts with target proteins is a major goal toward ultimately illuminating how OGT engages in protein complexes and selects its protein substrates. In addition, identifying how full-length OGT assembles may provide insights into how mutations contribute to disease. Difficulties in obtaining a full-length structure of OGT, however, has led to structural insights derived from only truncated proteins, initially a dimeric 11.5 TPR-only construct (TPR 1–11.5) and more recently the catalytic domains appended to TPR10-13.5 (Fig. 1a, b)^13,14^. The former demonstrated the TPR region generates an elongated superhelix with an internal groove lined by an asparagine ladder^10,14^. These asparagine residues are involved in the selection and binding of OGT substrates by making bidentate hydrogen bonds with the peptide backbone^9^. Recent work has also demonstrated aspartate residues which line the TPR lumen which are involved in peptide selection^15,16^. Originally OGT was proposed to form a trimer in solution, but the crystal structure of the TPR 11.5 construct suggested OGT dimerised around an interface formed at TPR 6-7^14^. This structural observation was supported by size-exclusion chromatography analysis, wherein disruptive mutation of residues lining the dimer interface reduced the size of the TPR 11.5 construct by roughly half^14^. Mutating these same residues in the full-length protein led to a modest decrease of in vitro activity. This reduction in activity suggests the dimer arrangement to be important either for protein stability or supporting catalysis in the full-length structure.

Since the current full-length model of OGT is a composite of two independently-solved structures, in this work we determine the structure by cryo-EM of full-length unliganded OGT to assess how the catalytic section is oriented with respect to the full 13.5 unit TPR region. Further, we provide an accurate understanding of the multimeric arrangement of OGT, which should aid in exploring how it complexes with large multi-protein partners^6,17,18^.

## Results

### Structure of full-length OGT

Through iterative cycles of particle classification in Relion we were able to resolve substantial heterogeneity in the particles and determine a 5.3 Å cryo-EM map (Supplementary Figs. 1–3)^19^. Into this map we could readily dock two copies of the X-ray structures of the catalytic region and most of the TPR 11.5 section (Fig. 1c, d). We find that OGT, in agreement with the 11.5 TPR-only crystal structure,^14^ forms a dimer supported by a relatively small interface (~560 Å^2^), which buries <1% of the total surface area of the full-length protein (Fig. 1).

Early maps generated during data processing provided density into which only the catalytic domain appended to TPR units 6-13.5 could be modelled, TPR units N-terminal of the dimer interface (TPRs 1–5) exhibited high heterogeneity, suggestive of flexibility in the TPR units of this region. Inspired by the work focussed on resolving structural heterogeneity in the catalytic domain of human γ-secretase, we attempted to capture some of the conformational states adopted by the N-terminal TPR units by careful classification of particles^20^. To search the volume around the missing N-terminal TPR repeats, a density map was created using molmap in Chimera from the atomic coordinates of the 11.5 TPR units crystal structure, which had been fitted into our map and localised to the poorly resolved N-terminal region^21^. This density map was used to create a mask (binary map extension of 3 pixels and a soft edge of 12 pixels) through Relion to search a large volume where the TPRs domains might be found (Supplementary Fig. 3)^19^. The generated mask was used to subtract signal from the regions of OGT already modelled using the Relion particle subtraction program^19^. This allowed us to perform careful clustering of the particles to identify TPR conformations within the masked section. Interestingly, we captured only one dominant conformation for this TPR region into which we could further model TPRs 3-5 for one chain of the dimer (Fig. 1e). We note that although the TPRs are flexible in this region, which may relate to their function, the dominant state we observed generally aligns well with the previous TPR 11.5 units crystal structure chain A (RMSD of 1.40 Å, residues 79–399)^14^.

### Confirming the dimeric state

**Fig. 2:**
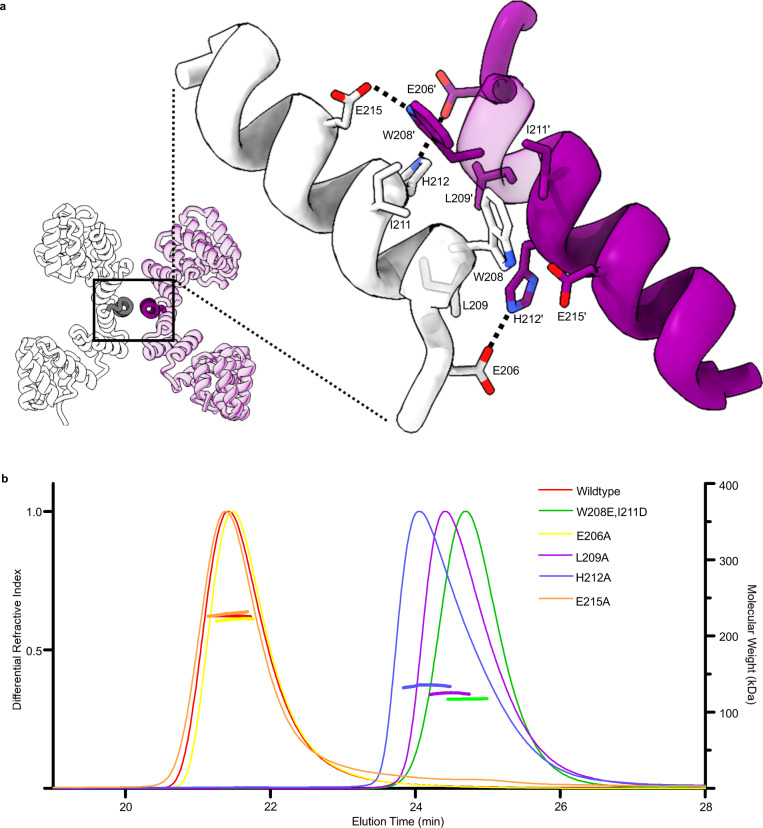
Probing the dimeric state. **a** The crystal structure of the 11.5 TPR construct with helices presented in expanded view depicted in darker shades. Enlarged view with residues subject to mutagenesis depicted. Dashed lines indicate hydrogen bonding. Purple and white denote separate chains, which form the dimer **b** SEC-MALLS trace demonstrating the multimeric state of OGT. Differential refractive index value depicted as a single continuous trace across the X-axis, while molecular weight estimates shown just for the region analysed. Source data are provided as a Source Data file.

Previously the dimeric state of OGT has only been inferred from size-exclusion chromatography (SEC) using the 11.5 TPR construct^14^. Owing to its elongated and non-globular shape, the wildtype 11.5 TPR construct migrates at an estimated molecular weight of ~120 kDa while only ~70 kDa in size^14^. Confirmation of the dimer assembly is realised by mutagenesis of two residues at the dimer interface leading to OGT migrating at ~70 kDa^14^. However, the large difference in size between actual and expected migration of OGT, the very small dimer interface, which promotes this interaction, and the full-length OGT construct having never been analysed leads to some ambiguity on the multimeric state of OGT in solution. To reinforce our cryo-EM observation of OGT forming a dimeric arrangement, the disrupting single mutants: E206A, L209A, H212A and E215A, and the double mutant matching that previously used to assess OGT dimer state (W208A, I211A) were produced. Owing to poor resolution at the cryo-EM model dimer interface, it was necessary to use the 11.5 TPR crystal structure to design these mutants (Fig. 2a). Mutants were assessed for multimeric state through size-exclusion chromatography multi-angle laser light scattering (SEC-MALLS; Fig. 2b). Wildtype, E206A, and E215 constructs were determined to be 226, 222, and 229 kDa, respectively, all fitting a dimer model (expected molecular weight, 235 kDa). The E206A and E215A mutants maintaining the dimeric state indicate that the loss of hydrogen bonding (E206 to H212 and E215 to W208) is tolerated in OGT and does not play as essential role at the interface. However, a minor peak for E215A detected at 25 min may indicate a very small sub-population of monomeric OGT, however due to the small quality of protein at this later peak and the tailing of the major peak, an accurate estimate of size cannot be reliably obtained. In contrast, the double mutant (W208A, I211A), and the single variants L209A, and H212A are monomeric at 117, 125, and 135 kDa, respectively (estimated monomer Mw is 117 kDa; Fig. 2). The brute force approach of the double mutant, through introduction of negatively charged amino acids facing one another likely represents the true monomeric state and agrees most strongly with the estimated monomer molecular weight. Unexpectedly, the L209A and H212A mutants break the interface but the earlier elution profile compared to the double mutant may suggest transient dimer formation. These results indicate that the hydrophobic network opposed to the polar interactions at the interface are the primary drivers of dimerization and that the interface provided by the TPRs is sufficient to support the assembly.

### Testing the activity of the dimer disrupting mutants

**Fig. 3:**
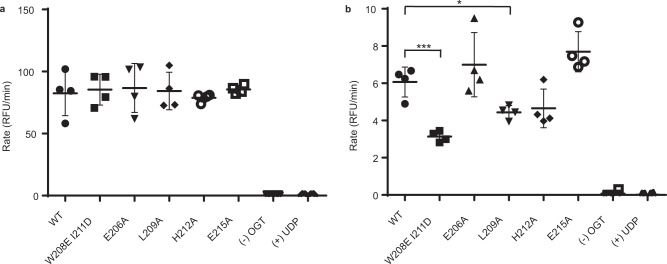
Transferase activity of the dimer disrupting mutants. **a** OGT wildtype and mutants were tested for their catalytic activity against HCF-1 peptide using 3 µM BODIPY-UDP-GlcNAc, 10 µM HCF-1 peptide, and 20 nM OGT. **b** OGT wildtype and mutants were tested for their catalytic activity against TAB1 protein in the presence of 10 µM BODIPY-UDP-GlcNAc, 0.6 µM TAB1, and 100 nM OGT. Measurements were performed in quadruplicate and yielded similar data in two biological replicates. Error bars represent one standard deviation (SD) from the mean. *P = 0.0101, ***P = 0.0005; two-tailed *t*-tests. Source data are provided as a Source Data file.

To test whether the dimer disrupting mutants affect the catalytic activity of OGT we conducted a series of kinetic assays using a fluorescent activity assay for OGT^22^. We first evaluated the activity of wildtype and mutant OGT towards glycosylation of a 26 amino acid peptide substrate derived from host cell factor 1 (HCF-1) using previously established conditions. Notably, we observed no significant differences in the rate of glycosylation of this substrate within the series (Fig. 3a). We hypothesised that the disruption of the dimer interface may play a greater role in the glycosylation of larger protein substrates that bind within the TPR lumen of OGT. To investigate this, we therefore modified the fluorescent activity assay to evaluate the glycosylation of recombinant TGF-beta activated kinase 1 (TAB1) protein, a known protein substrate of OGT^23^. In contrast to the shorter HCF-1 peptide substrate, using TAB1 protein as a sugar acceptor reveals significant differences between the glycosyl transfer catalysed by the mutants as compared to wildtype OGT (Fig. 3b). Strikingly, the double mutant shows the least activity as manifested in a relative second order rate constant that is ~50% lower as compared to wildtype (Supplementary Fig. 6). These results collectively show that mutations affecting the dimeric state of OGT do not impact the catalytic activity of OGT toward peptide substrates but rather influence activity on protein substrates that engage the TPR domain of OGT. These data also suggest that the ability of OGT to dimerise may be an important factor governing OGT activity within cells.

### Comparison of the cryo-EM model to X-ray crystallography structures

**Fig. 4:**
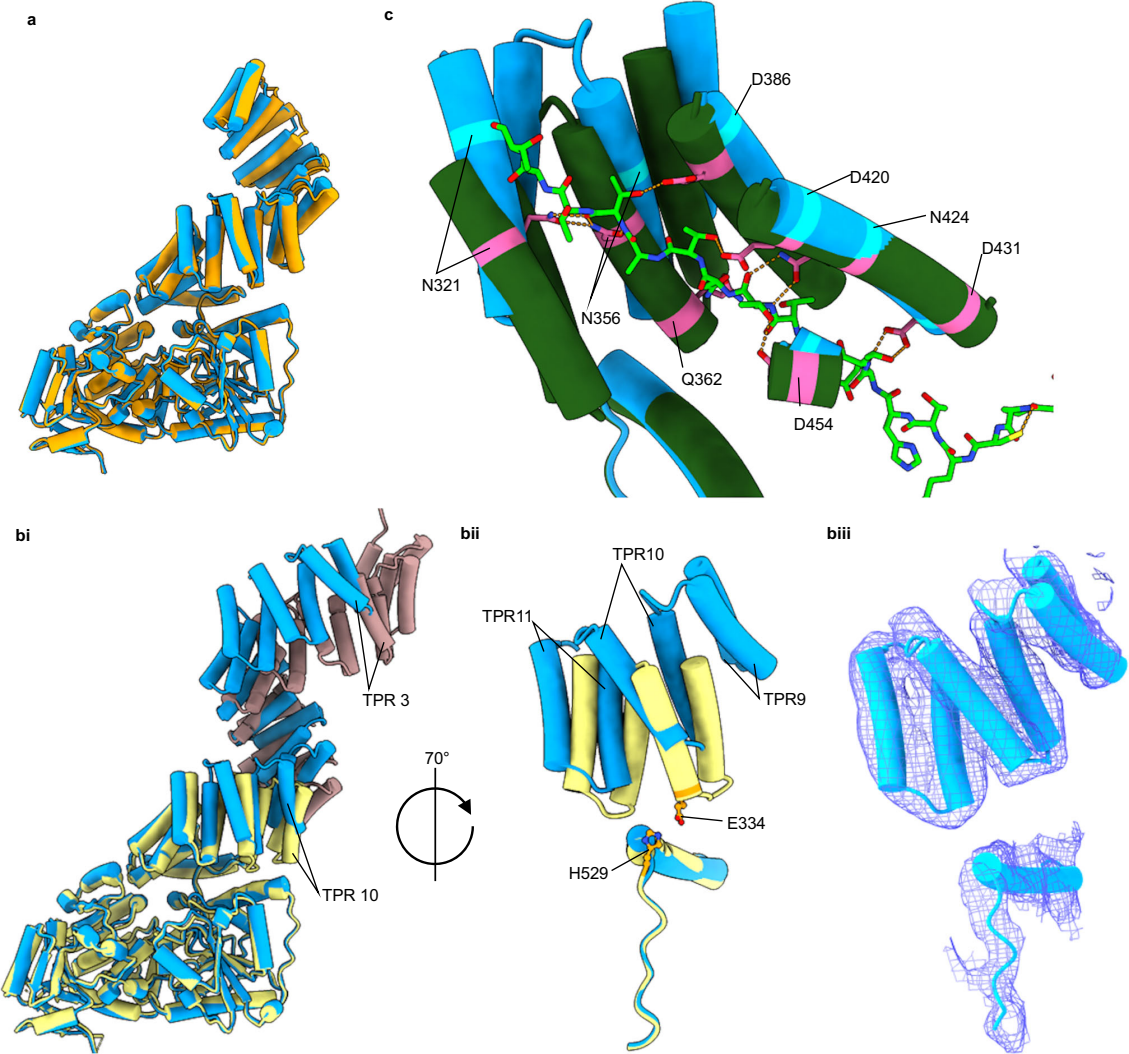
Comparison of cryo-EM model to crystal structures. **a** Superimposition of chain A (blue) onto chain B (orange) of the 5.3 Å structure demonstrates the distortion of the twofold symmetry. **bi** Comparison of the cryo-EM model (chain A, blue) to the OGT/UDP X-ray structure with the TPR 11.5 X-ray structure (chain A) superimposed (PDB: 3PE3, yellow. PDB: 1W3B, pink). Residues 544–1028 were used for superimposition. **bii** TPRs 9–11 and their relative position to residues 505–538 of catalytic region. Residues suggested to promote TPR 10 anchoring to the catalytic region depicted in orange. **biii** Cryo-EM map into which the TPRs and residues 505–538 were modelled. **c** Comparison of cryo-EM model (chain A) to the OGT/UDP/HCF-1 peptide ternary complex (PDB: 4N3C, green)^9^. Residues 544–1028 were superimposed. Hydrogen bonding interactions depicted as dashed orange lines.

The 11.5 TPR crystal structure forms a pseudo-symmetric twofold dimer, with C-terminal TPR units adopting different conformations due to a 40° bend seen in one chain between TPR units 9 and 10 (Fig. 1aii)^14^. This kink was previously suggested to be due to crystal contacts causing chain B to bend^14^. Notably, by cryo-EM we do not observe this 40° kink but find that, in the absence of crystal contacts, OGT does deviate from the previously observed 2-fold symmetry with each chain being related to one another by a 173° rotation. This difference arises from variation in the TPR positions at the dimer interface that is propagated through the structure (Fig. 4a). It is unclear whether the deviation away from a perfect 2-fold symmetry is related to a yet undiscovered function of OGT.

Our cryo-EM generated model superimposes well onto the catalytic region of the OGT/UDP X-ray structure (PDB: 3PE3)^13^, suggesting that binding of UDP/UDP-GlcNAc causes no conformational changes that disrupt the conformation of the catalytic domains (Fig. 4b). Structural variations between the cryo-EM and crystal-based models therefore exclusively relate to the positioning of the TPR regions with respect to the catalytic section. Crystal structures of UDP bound OGT suggest TPR 10 is anchored to the catalytic region through bonding of E334 to H529, whereas molecular dynamic simulations have suggested this interaction is transient with the TPR region having the capacity to “open” though a hinge like motion to permit active site access^13^. We note from our cryo-EM structure that TPR 10 is shifted 5 Å further away from the catalytic domain compared to the OGT/UDP X-ray structure (Fig. 4b, E334 Cα to H529 Cα comparison). Since the catalytic domains superimpose well between the OGT/UDP structure and our structure, we speculate this conformation would also be adopted in both the UDP and UDP-GlcNAc bound OGT structures.

The positioning of TPR 10 in the cryo-EM model widens the gap between the TPR region and the catalytic region compared to the previously proposed full-length model and leads to a propagation of increasing discrepancy in the positions of the TPR units as they extend away from the catalytic domain toward TPR 1 and the N-terminus (Fig. 4b). The position of TPR 3, for example, deviates by ~14 Å with respect to the catalytic region between the composite model and the cryo-EM model, whereas this distance for TPR 10 is only 5 Å. While we do observe some flexibility in the TPR units, as evident from chain A and B deviating slightly in arrangement, throughout data processing we did not observe significantly different conformations of the TPRs from our model, suggesting the dominant conformation of TPR 10 does not position itself closer to the catalytic region when OGT is unliganded (Fig. 4a).

**Fig. 5:**
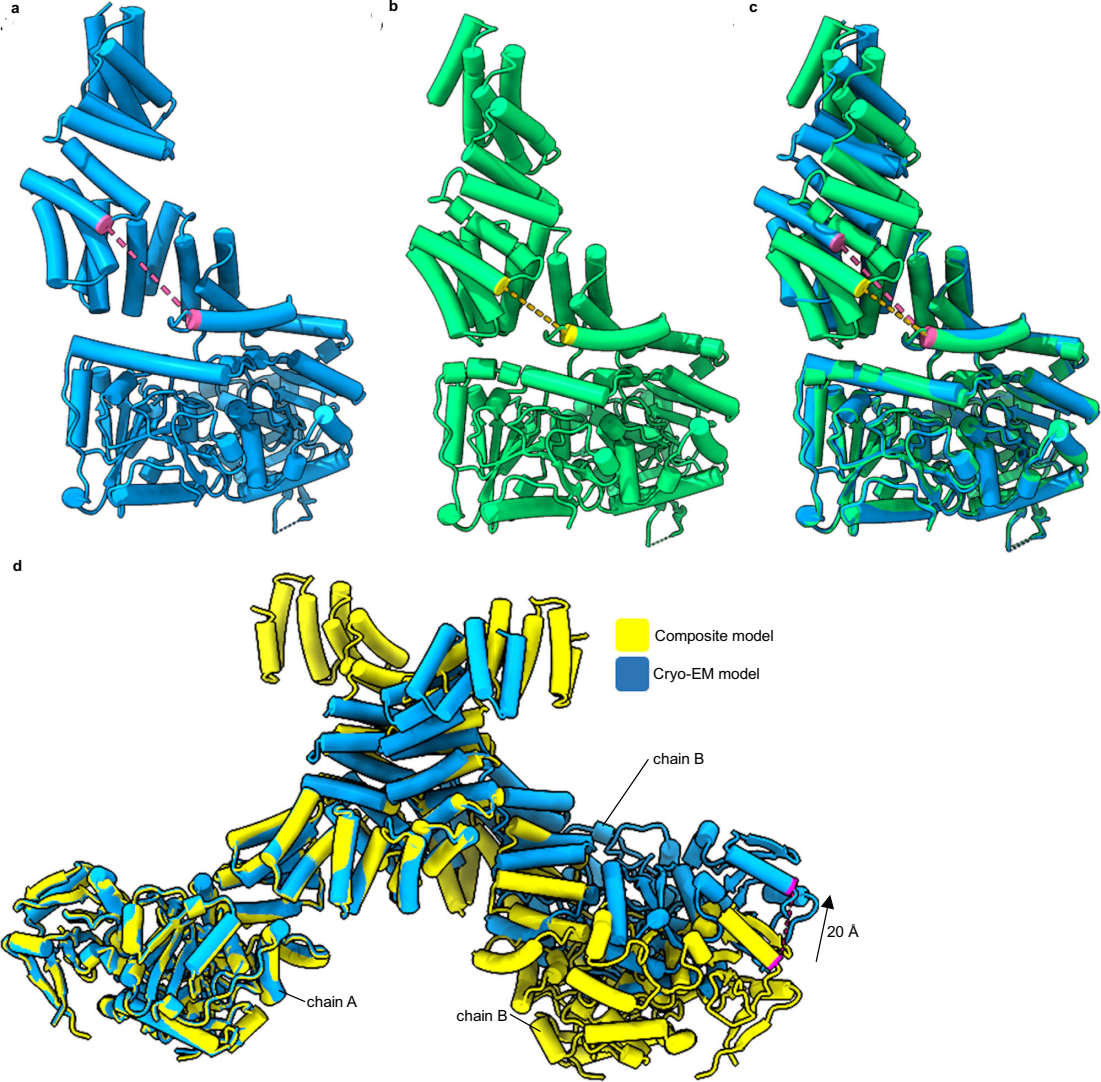
Deviations from proposed full-length models of OGTs. **a** Cryo-EM model of OGT with the measured distance between V229 Cα to E488 Cα depicted as a pink dashed line. **b** Crystal structure composite model of OGT generated by superimposition of residues 313–400 of 11.5 TPR chain A (PDB: 1W3B) onto the OGT/UDP structure (PDB: 3PE3)^13, 14^. Distance between V299 Cα to E488 Cα depicted as a yellow dashed line, calculated as 25 Å for **a** and 18 Å for **b**. **c** Superimposition of residues 544–1028 of the Cryo-EM model onto the speculated model. A large face is opened in our structure compared to the composite structure. **d** Superimposition of chain A of the L254F composite model onto chain A of our cryo-EM structure^24^. Composite model was generated by superimposing our catalytic domain, with the correct TPR 10 position, onto the 11.5 TPR L254F mutant dimer crystal structure (PDB: 6EOU) using residues 260–360^24^. A movement of 20 Å would be required in chain B for equivalent T771 Cα atoms to overlay.

X-ray structures of OGT with a HCF-1 peptide bound to TPR units 10-13.5 demonstrate distinct bidentate hydrogen bonding interactions coordinating the peptide backbone^9^. If the OGT/HCF-1 crystal structure is correct regarding placement of TPR units 10-13.5, the unliganded state would need to undergo significant conformational changes for residues to make these same peptide interactions (Fig. 4c). In doing so, the TPR units would move closer to the catalytic domain, limiting the accessible space to the TPR groove. In the unliganded structure, the immediate advantage of having a larger gap between TPR units and the catalytic domain would be for protein substrates to have increased access to both the TPR groove and catalytic cleft (Fig. 5a–c). This effect is more pronounced in our structure compared to the previously proposed composite model with the hypothesised distance between TPR 9 and the transitional helix being 18 Å in the composite model, and 25 Å in the cryo-EM model (Fig. 5a–c).

In addition, to increasing access to the TPR lumen, the position of TPR 10 in conjunction with the dimeric state of OGT may allow us to infer and model how OGT mutations cause disease. For instance, an XLID causing mutation (L254F) in the TPR region has been demonstrated to distort the TPR superhelix^24^. Comparing the L254F dimer arrangement to our cryo-EM model, it is evident that this mutation also causes severe perturbation to the dimer assembly (Fig. 5d), suggesting a mechanism by which such mutations may contribute to disease is by altering interactions of OGT with some of its many partners or substrates. Our model does not provide further mechanistic insights into how other TPR localised XLID mutations (A259T, R284P, A319T and E339G) contribute to disease since structures for these mutants are not available. Prior work, using the 11.5 TPR construct has suggested that all XLID TPR mutants are dimeric in solution, but it may be possible that the dimeric state is altered for these mutants as we suggest may be the case for L254F here^25^.

## Discussion

**Fig. 6:**
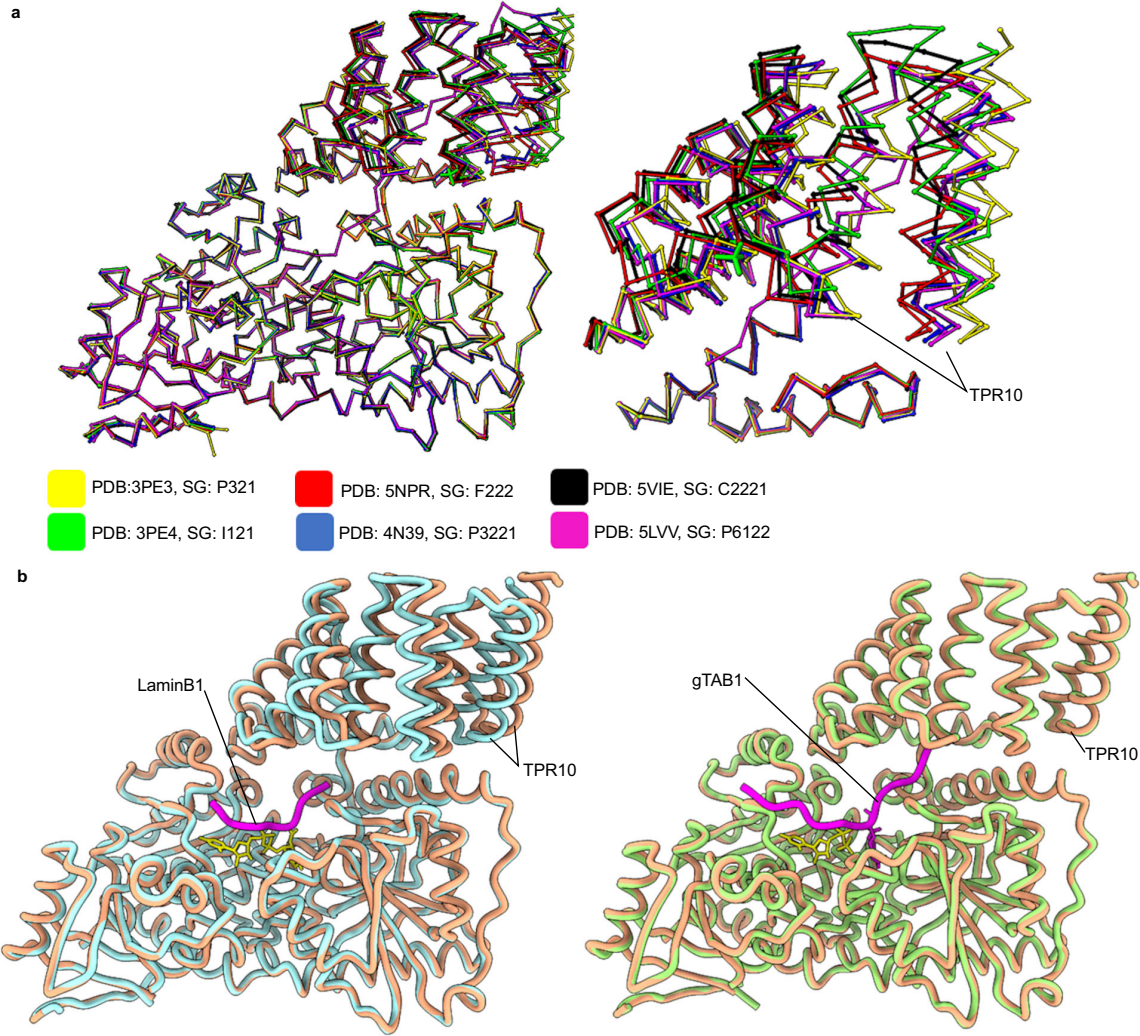
Crystal packing changes the conformation adopted by TPR10. **a** Superimposition of OGT structures (polypeptide backbone only) onto OGT/UDP (PDB: 3PE3, left) and the position adopted in TPR10 of proteins within the space group (right)^9, 13, 35–37^. No difference in the catalytic regions are observed, while TPR 10 is susceptible to crystal packing due to a high degree of flexibility. **b** In the OGT/UDP-5SGlcNAc/LaminB1 peptide ternary complex (SG: F222, PDB: 5BNW, blue) the peptide (purple) does not interact with the TPRs, however, TPR 10 is shifted in relation to OGT/UDP structure (SG: P321, pink)^27^. The OGT/UDP/gTAB1 ternary peptide structure (SG: P321, PDB: 4AY5, green) shows the peptide (purple) extending into TPR units and TPR 10 is identical compared to the OGT/UDP structure (SG: P321, pink)^28^.

As of early 2021, the OGT field has been greatly aided by the release of 35 structures of the catalytic domain of OGT bound to a myriad of substrates, peptides, and inhibitors. This has enabled careful dissection of catalysis in the active site. In general, our model reaffirms observations previously noted, including a tight match to the catalytic domain OGT, and adopting a dimeric assembly supported by a relatively small interface^14^. We note that without crystal formation, TPR units N-terminal of the dimer interface exhibit considerably conformational heterogeneity and could only be partly resolved by particle subtraction, hinting that flexibility in this section may play a role in how OGT selects larger substrates. Indeed loss of asparagine residues in the TPR 3-6 segment leads to decreases in glycosylation^16^. Major differences in our model relate to how the TPR units position themselves in respect to the catalytic region. Explaining the variations in our observations from the crystal structures of OGT with UDP, UDP-GlcNAc, and UDP-5SGlcNAc (PDBs: 3PE3, 4GZ5 and 4GZ6, respectively), we note these were all solved in the same space group (SG) and pack with two further copies of OGT that likely cause compression of TPR 10 towards the catalytic domain^13,26^. Indeed, crystal packing and space group appear to be the primary drivers of the TPR conformations seen in crystal structures (Fig. 6a). For example, in the OGT/UDP-5SGlcNAc/LaminB1 peptide ternary complex (SG: F222) the peptide does not interact with the TPRs yet a 6 Å shift in TPR 10 is seen as compared to the OGT/UDP structure (SG: P321), suggesting the altered position is attributable to different space-groups (Fig. 6b)^13,27^. In agreement, the OGT/UDP/gTAB1 ternary peptide structure (SG: P321) shows the peptide extending into the TPR units yet no change occurs in TPR 10 as compared to the OGT/UDP structure (SG: P321), again suggesting that positioning of TPR 10 is a result of crystal packing rather than ligand binding (Fig. 6b)^28^.

In summary, previous studies have used the superimposition of the X-ray derived models of the TPR 11.5 construct onto the TPR 4.5 construct to represent full-length OGT protein. We here demonstrate, however, that this superimposition model is unlikely to represent the dominant conformational state. In addition, we illuminate a dimeric assembly unfettered by crystal contacts that suggests XLID causing mutations in the TPR region cause distortion of this dimeric conformation. We further show the importance of the dimeric state of OGT for glycosylation of a protein substrate using kinetic assays. This work represents the first study of OGT by cryo-EM, and we expect further work in this direction will continue to shed light into how the TPRs bind and co-ordinate interactions with both protein partners and substrates.

## Methods

### Expression and purification of OGT for cryo-EM analysis

Full-length OGT cDNA (isoform 1, UNIPROT identifier: O15294-3) in a pET28a plasmid with N-terminal 6xHis-tag and thrombin cleavage site was transformed into BL21-Gold(DE3) *Escherichia coli* cells for expression. Residue numbering follows the sequence register of isoform 1 and thus the convention used for the OGT4.5 structures. ID mutants follow the isoform 3 numbering system and are 10 amino acids out of register with our structure. These cells were grown in 4 l of Terrific Broth (TB) to an OD_600_ of 1.8–2.0 before being cooled at 4 °C for 30 min. Cultures were then induced with 0.2 mM IPTG and incubated in a shaking incubator at 16 °C overnight. Cells were collected by centrifugation at 5400 × *g* for 20 min. The cell pellet was resuspended in 100 ml of buffer A (50 mM HEPES 7.4, 500 mM NaCl, 20 mM imidazole, 1 mM DTT) supplemented with 1 mM AEBSF, DNAse 1 and lysozyme. Lysis of cells was by cell disruptor before clarification by centrifugation at 41,700 × *g* for 1 h. Lysate was passed over 2 × 1 ml HisTrap FF columns (GE Healthcare) equilibrated in buffer A. Protein was eluted by a stepwise gradient of buffer B (50 mM HEPES pH 7.5, 500 mM NaCl, 250 mM imidazole, 1 mM DTT) in buffer A. Fractions containing protein were dialysed against 1 l of 25 mM HEPES 7.4. 150 mM NaCl and 1 mM DTT overnight at 4 °C. Protein precipitate was removed by centrifugation before protein was concentrated to 2 ml using a 10,000 MWCO Vivaspin® spin-concentrator (Sartorius). The concentrated sample was size excluded on a 16/60 Superdex 200 column (GE Healthcare) in 25 mM HEPES pH 7.5, 150 mM NaCl and 1 mM DTT. Clean fractions of protein were concentrated to 1.2 mg/ml and snap frozen.

### Expression and purification of OGT for SEC-MALLS analysis

To improve on protein yield, codon optimised full-length OGT DNA (isoform 3, UNIPROT identifier: O15294-1, insertion of 10 amino acids between positions 13–22 compared to isoform 1) was inserted into a pET28a vector with C-terminal 6xHis-tag. Mutants were produced through standard site-directed mutagenesis protocols. Constructs were confirmed by sequencing of the full open-reading frame of OGT. Expression and His-tag purification steps were as described above except only 2 l of TB was required for each mutant. Instead of dialysis, protein was diluted to 50 mM NaCl with 20 mM HEPES 7.5 and loaded onto a 5 ml Q Sepharose FF column pre-equilibrated in buffer C (25 mM HEPES 7.5, 50 mM NaCl and 1 mM DTT). Protein was eluted by a stepwise gradient of buffer D (25 mM HEPES 7.5, 1 M NaCl and 1 mM DTT) in buffer C. Fractions containing pure protein were pooled and concentrated to 2 ml using 10,000 MWCO Vivaspin® spin-concentrators (Sartorius), before being size excluded on a 16/60 Superdex 200 column (GE Healthcare) in 25 mM HEPES pH 7.5, 150 mM NaCl and 1 mM DTT. Protein was concentrated to 3 mg/ml and snap frozen.

### Cryo-EM sample preparation and data collection

For cryo-grid preparation, OGT was diluted in 25 mM HEPES pH 7.5, 150 mM NaCl and 1 mM DTT to 1 mg/ml and supplemented with 0.5% glycerol. The cryo-EM specimen was prepared using a FEI Vitrobot mark IV set at 100% humidity with a blot time of 4 secs and blot force of -10 arbitrary units. A 2 µl aliquot of OGT was applied to a UltrAuFoil R1.2/1.3 300 mesh gold grid that had been glow-discharged for 3 min at 20 mAmp/0.38 mBar using the ambient atmosphere. The grid was then blotted and plunge-frozen in liquid ethane. Cryo-EM images were acquired on a Gatan K3 detector in super-resolution mode using a 300 kV FEI Titan Krios microscope at the Electron Bio-Imaging Centre, UK. EPU (Thermo Fisher Scientific) was used to automate data collection. Fifty movie frames were collected at a nominal magnification of x130,000 (which creates a calibrated super-resolution pixel size of 0.3225 Å). The dose was 1.04 e/ Å^2^ per frame with a total accumulated dose of 52 e/ Å^2^. A 100-micron objective aperture was used. Defocus of micrographs ranged from -1.8 microns to -3.2 microns. Autofocus was run every 5 microns. A total of 9233 micrographs were collected.

### Micrograph correction and auto-picking procedures

We used MotionCor2 within Relion-3.0.5 for frame motion correction and CTFFIND-4.1 for estimating the contrast transfer function parameters^19,29,30^. A subset of 99 micrographs were picked from using the Laplacian-of-Gaussian (LoG)-based auto-picking procedure in Relion-3.0.5^19^. Picked particles were used to generate 2D class templates for subsequent particle picking from all micrographs. All templates were low pass filtered at 20 Å to prevent the introduction of bias.

### Data processing and generation of maps

Auto-picked particles (4.32 × 10^6^ particles) were 2D classified through Relion-3.0.5, after discarding poor particles the total number of particles was reduced to 1.53 × 10^6^.^19^ Evidently some 2D classes appeared to represent the hypothesised dimeric structure of OGT, however due to the elongated shape of OGT we were unsure if some of the smaller classes were OGT or noise (Supplementary Fig. 1). We decided to take a cautious approach of including these particles in downstream steps and using 3D classification steps to remove poor particles. Owing to the large number of particles and the associated computation time, we were required to do this in several smaller steps. Initially we extracted the particles downsampled to 5.208 Å and used an angular sampling of 7.5°, this removed the bulk of the poor particles (Supplementary Fig. 2). We then performed a series of further 3D classification jobs, incrementally reducing both the pixel size and the angular sampling (Supplementary Fig. 2). This reduced the number of particles to 534,051. Throughout these classifications, one class was dominant with other particles falling into either noise or poorly resolved forms of this dominant class (Supplementary Fig. 2). At map threshold levels where density of the catalytic domain helices remained distinct from one another, it was impossible to observe the TPRs N-terminal of the dimer interface, likely because of significant structural heterogeneity in this region. Reducing the viewing threshold of the map to near that where background noise is visible did yield some density indicative of the missing N-terminal TPR repeats. Initially we tried to obtain density for these TPR repeats by limiting the resolution E-step to 8 Å during 3D classification to prevent overfitting. In doing so, one of the four classes displayed improved density for the N-terminal TPRs (Supplementary Fig. 3). We also tried increasing resolution by refining the CTF of the particles and performing Bayesian polishing through Relion, however no increase in resolution was obtained^19^. Particles with signal subtraction (see Results) were 3D classified into four classes with particle alignment based on a previously refined structure. Only one class (74102 of 329,567 particles) provided traceable density and this density correlated well to the expected shape of the N-terminal TPR region (Supplementary Fig. 3). From this single class, the subtracted particles were reverted to their non-subtracted form from this single class and refined. Following post-processing, a 5.3 Å map was generated (Fourier shell correlation (FSC) cutoff of 0.143)^31^ with good connectivity for almost the full polypeptide in one chain of the dimers. Into this density it is possible to trace the full catalytic domain and TPRs 3-13.5, albeit at low resolution in the most N-terminal segments (Supplementary Fig. 4).

### Modelling OGT

A composite model of full-length OGT was produced by superimposing overlapping residues of OGT/UDP (PDB: 3PE3)^13^ and the TPR 11.5 (PDB: 1W3B, chain A)^14^. Using Chimera’s “fit in map” function we positioned two copies of the OGT/UDP structure (PDB: 3PE3) into the density map before using these as superimposable templates to fit the full-length structure using “matchmaker”^21^. This was necessary to overcome the bias introduced by the incorrect conformation of the TPRs when attempting to fit the full-length structure into the map. To correct the conformation of the TPR repeats, iterative cycles of Flex-EM and manual adjustment in *Coot* were undertaken^32,33^. Final refinements were carried out in Phenix with rigid body restraints^34^. The resolution was too poor to observe correct side chain rotameric states (although these were used in rigid body refinement), thus we have submitted our model without sidechains. Figures were produced using Chimera and ChimeraX^21^.

### Size-exclusion chromatography multi-angle laser light scattering (SEC-MALLS)

SEC-MALLS analysis was conducted on a system comprising a Wyatt HELEOS-II multi-angle light scattering detector and a Wyatt rEX refractive index detector linked to a Shimadzu HPLC system (SPD-20A UV detector, LC20-AD isocratic pump system, DGU-20A3 degasser and SIL-20A autosampler). Experiments were conducted at room temperature. A Superdex S200 10/300 GL column pre-equilibrated in running buffer (20 mM HEPES 7.5, 200 mM NaCl) was used for separation. Sample injection was 100 µl of 3 mg/ml OGT. Flow rate was set at 0.5 ml/min. Shimadzu LabSolutions software was used to control the HPLC and Astra 7 software for the HELEOS-II and rEX detectors. The Astra data collection was 1 min shorter than the LC solutions run to maintain synchronisation. Data were analysed using the Astra 7 software and figures created using GraphPad Prism 5. MWs were estimated using the Zimm fit method with degree 1. A value of 0.182 was used for protein refractive index increment (dn/dc).

### OGT enzymatic activity assays

Enzyme kinetics were performed using a previously described fluorescent OGT activity assay with minor modifications^22^. All assays were carried out in black Nunc 384-well plates (262260, Thermo Fisher Scientific). For measuring glycosyl transfer to HCF-1 peptide, 20 nM OGT wildtype and mutants were incubated in the presence of 10 µM biotinylated host cell factor 1 (HCF-1) peptide (Biotin-VRVCSNPPCS*THETGTTNTATTATSN, Biomatik) and 3 µM BODIPY-UDP-GlcNAc in PBS containing 0.02% triton x-100, 12.5 mM MgCl_2_ and 1 mM DTT. The assay was incubated for 40 min at ambient temperature as the reaction rate was linear during this time frame for both HCF-1 and TAB1 assays (Supplementary Fig. 7). The reaction was terminated by addition of 10 mM UDP in PBS with 0.02% triton containing 0.17 mg/ml streptavidin-coated magnetic beads (M-1002, NanoLink, Vector Laboratories). To wash away non-transferred BODIPY-UDP-GlcNAc, the plate wells were washed using a BioTek EL406 plate washer containing a magnetic adapter and a BioTek 384 F magnet that allows for immobilization of HCF-1 - bead complexes (12 wash cycles). The fluorescence signal corresponding to glycosyl transfer to HCF-1 was measured using a Biotek Neo2 plate reader at 490 nm excitation and 525 nm emission. Measuring glycosyl transfer to TGF-beta activated kinase 1 (TAB1) protein was done in a similar fashion. TAB1 cDNA was purchased from OriGene (SC116337) and expressed with a 6xHis-tag using a pET28a vector. OGT wildtype and mutants were incubated at 100 nM in the presence of 0.2 to 0.6 µM his-tagged TAB1 protein and 10 µM BODIPY-UDP-GlcNAc in PBS containing 0.01% triton x-100 and 12.5 mM MgCl_2_.For immobilization of his-tagged TAB1, Nickel-NTA dynabeads (10103D, Thermo Fisher Scientific) were used and the wells were subjected to six wash cycles using buffer containing 50 mM Na_2_HPO_4_, 300 mM NaCl, 0.01% triton x-100 at pH 8. Four technical repeats were performed for each condition. Background fluorescence was measured in wells containing 0 µM TAB1, 0 µM HCF-1, or at 0 min incubation and was subtracted from experimental samples. Kinetic data were analysed using GraphPad Prism 5 and 6.

### Reporting summary

Further information on research design is available in the Nature Research Reporting Summary linked to this article.

## Supplementary information

Supplementary Information

Reporting Summary

## Data Availability

The cryo-EM maps and models generated in this study have been deposited in the PDB and EMDB databases under accession codes 7NTF and EMD-12588, respectively. Source data are provided with this paper for Figs. 2 and 3, and Supplementary Figs. 6 and 7. Raw cryo-EM micrographs are available from authors upon reasonable request. The PDB was used to access some entries used for comparisons in this work including PDBs IDs: 1W3B, 3PE3, 3PE4, 4N3C, 4N39, 4AY5, 4GZ5, 4GZ6, 5NPR, 5LVV, 5VIE, 5BNW, and 6EOU. Source data are provided with this paper.

## Source data

Source Data
