# Supplementary material for: Cryo-EM structure provides insights into the dimer arrangement of the O-linked β-N-acetylglucosamine transferase OGT
Source: Nat Commun. 2021 Nov 11;12:6508. doi: 10.1038/s41467-021-26796-6 (PMC8586251; doi:10.1038/s41467-021-26796-6)
Supplement: Supplementary file 1 — Supplementary Information [file 41467_2021_26796_MOESM1_ESM.pdf]

Supplementary Table 1. Statistics for Cryo-EM data collection and processing.

	5.3 Å OGT map (PDB:7NTF)
**Data Collection and processing**	
Magnification	130,000 x
Voltage (kV)	300
Detector	Gatan K3
Total electron exposure (e/Å^2)	52
Defocus range (μm)	-1.8 to -3.2
Pixel size (Å)	0.3225 (super-resolution)
Symmetry imposed	C1
Initial particle particles	4326360
Final number of particles	74102
Map resolution (Å)	5.32
FSC threshold	0.143
Map sharpening B-factor (Å^2)	-223
**Refinement (peptide backbone only)**	
Initial model used (PDB code)	1w3b and 3pe3
No. of atoms	
Protein	8607
R.M.S. deviations	
Bonds (Å)	0.004
Angles (°)	1.063
Fit to map (CC mask, Phenix)	0.67
Clash score	3.33
Ramachandran Favoured (%)	96
Ramachandran Allowed (%)	4
Ramachandran Outliers (%)	0

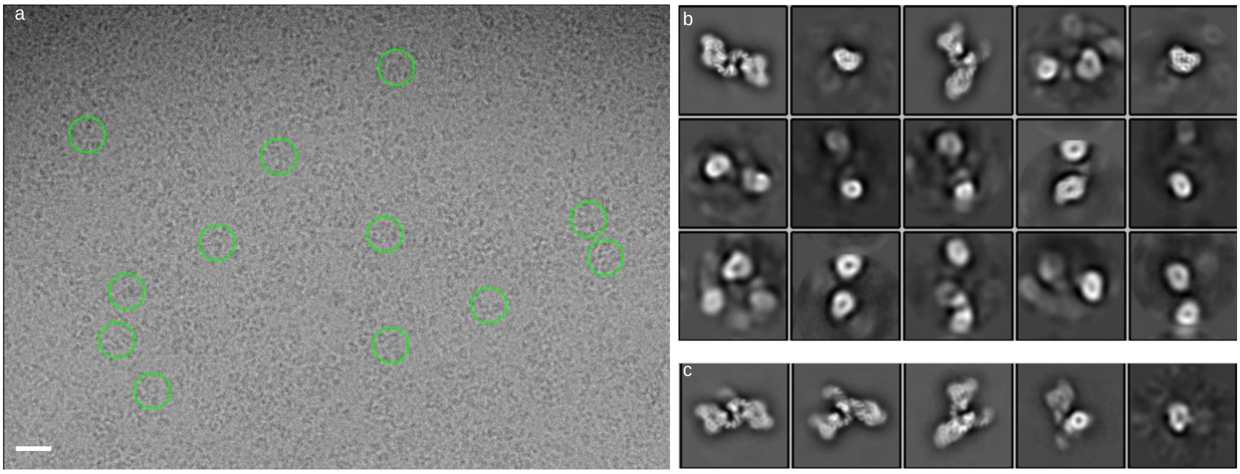

Supplementary Figure 1. Cryo-EM data processing. **(a)** Representative micrograph of OGT from a hole on a UltrAuFoil R1.2/1.3 300 mesh gold grid that had been glow-discharged for 3 mins at 20 mA/0.38 mBar. Micrograph is representative of 9233 micrographs from a single data collection. Scale bar is 200 Å. Green cycles indicate a few picked particles from the micrograph. **(b)** 2D class averages generated from auto-picked particles prior to 3D classification. **(c)** 2D class averages of the final refined particles.

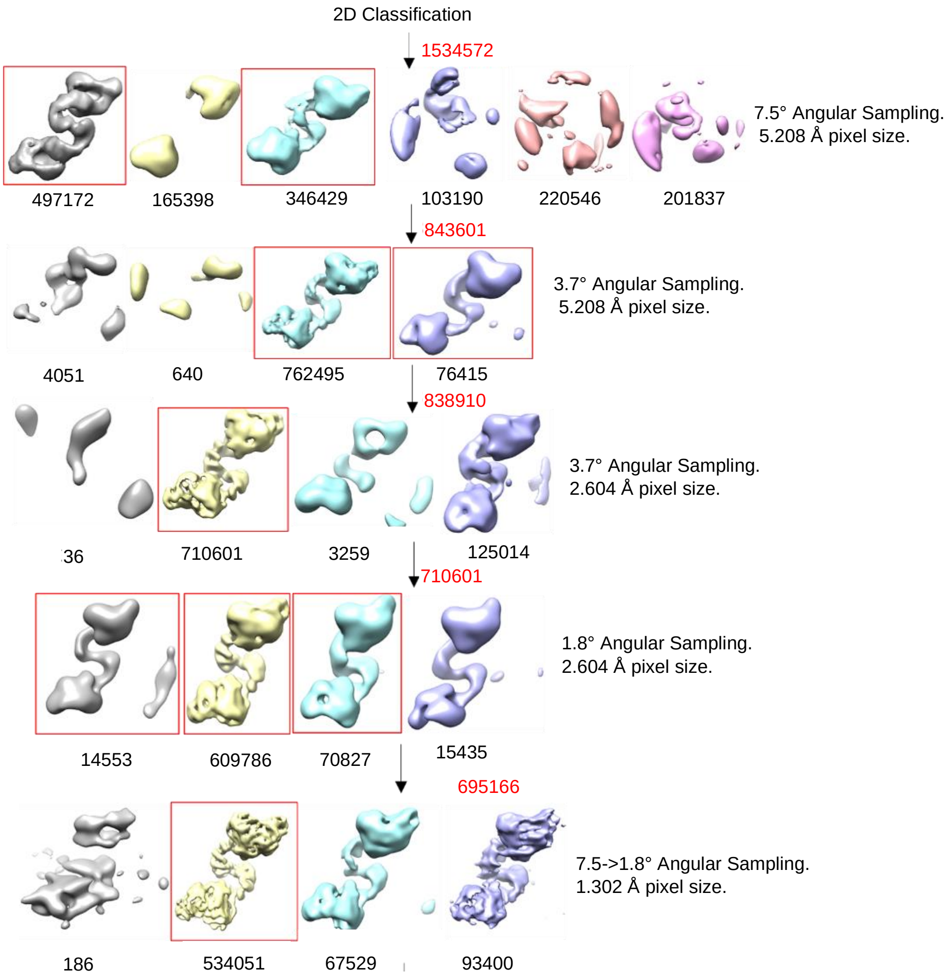

Supplementary Figure 2. Initial 3D classification steps taken to remove bad particles. Red boxes indicate classes selected for downstream processing and the total combined number of particles selected are written in red. Numbers in black below classes indicate the number of particles in the associated class. Discarded classes were either low-resolution, noisy reconstructions of a single prominent class common to all runs or background noise.

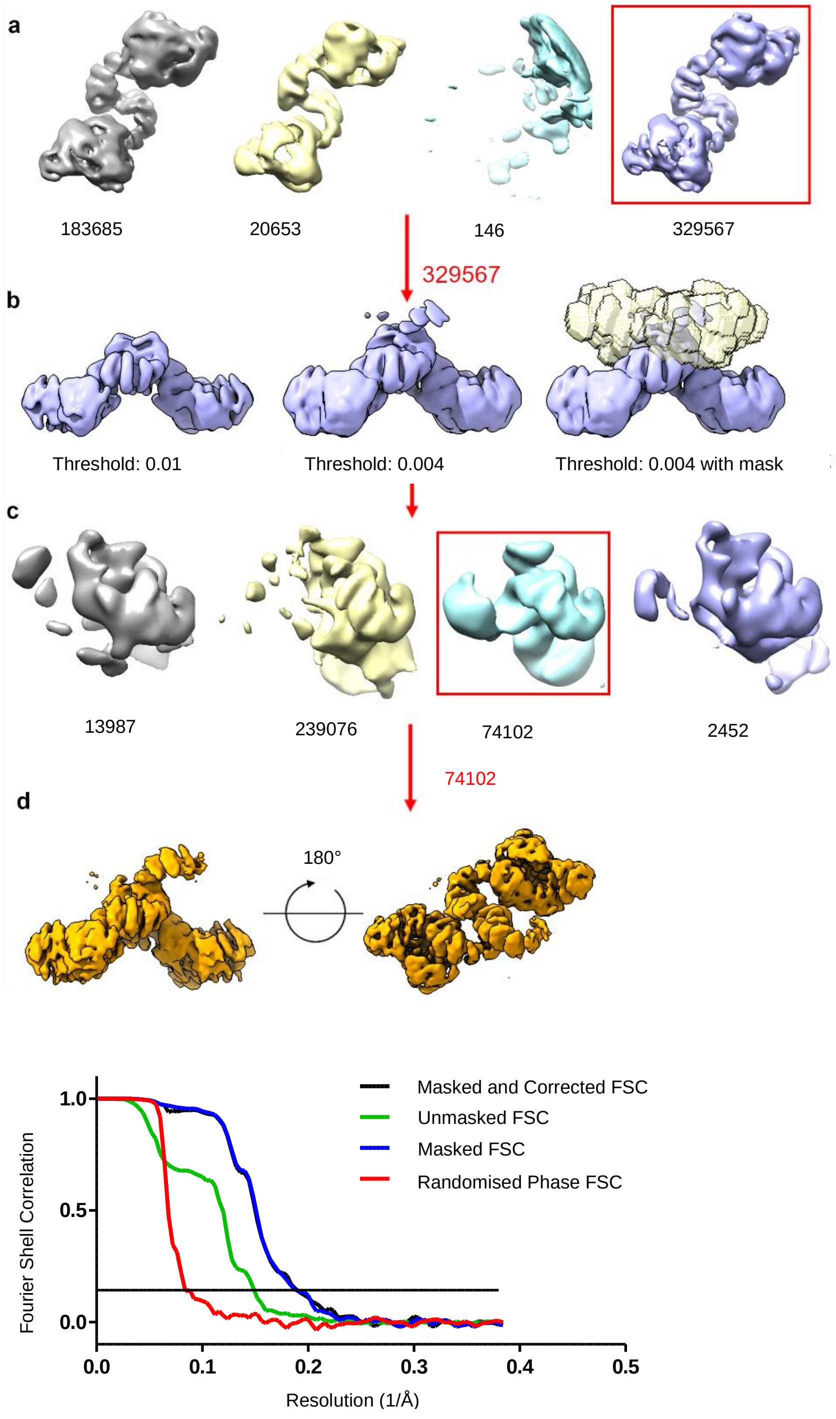

Supplementary Figure 3. Workflow for obtaining the 5.3 Å map. Red boxes and text indicate particles used for downstream processing. **(a)** 3D classification was performed on final particles from Supplementary Figure 2 but with the resolution E-step limited to 8 Å yielding a class with better defined density in the N-terminus of OGT. **(b)** At lower Chimera thresholds, density matching the TPRs at the N-terminus of Class 4 was evident. A mask was created (right) using the hypothesised full-length OGT structure. This mask was used to perform particle subtraction on the Class 4 particles. **(c)** Clustering of particles after the subtraction job yielded a clean class in which the density correlated to the missing TPRs. **(d)** Restoring and refining this subset of particles produced a 5.3 Å map in which we could model 11.5 TPR units. Horizontal black line, FSC=0.143.

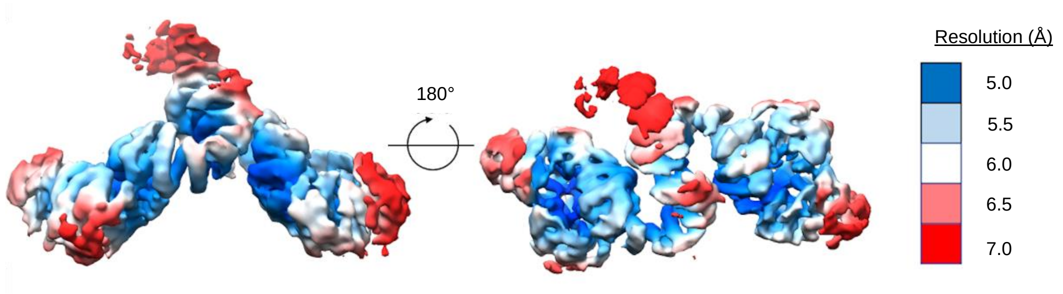

Supplementary Figure 4. Local resolution of the cryo-EM map. Local resolution estimate obtained through the Relion 3.0.5 local resolution job.

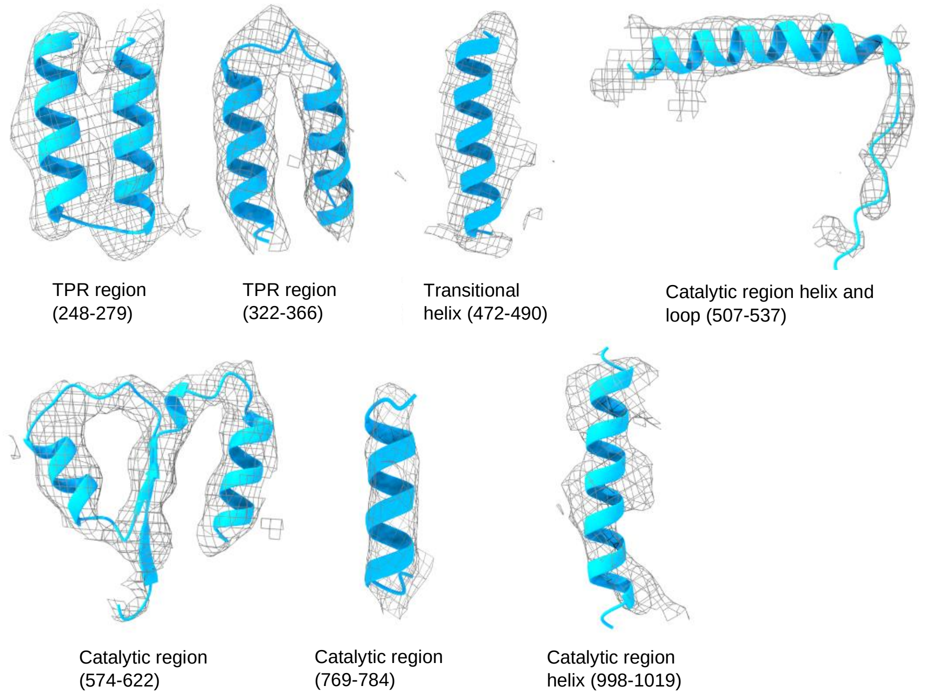

Supplementary Figure 5. Representative densities taken from different parts of the map of OGT (carved at 2.6 Å radius with a threshold of 0.02).

.

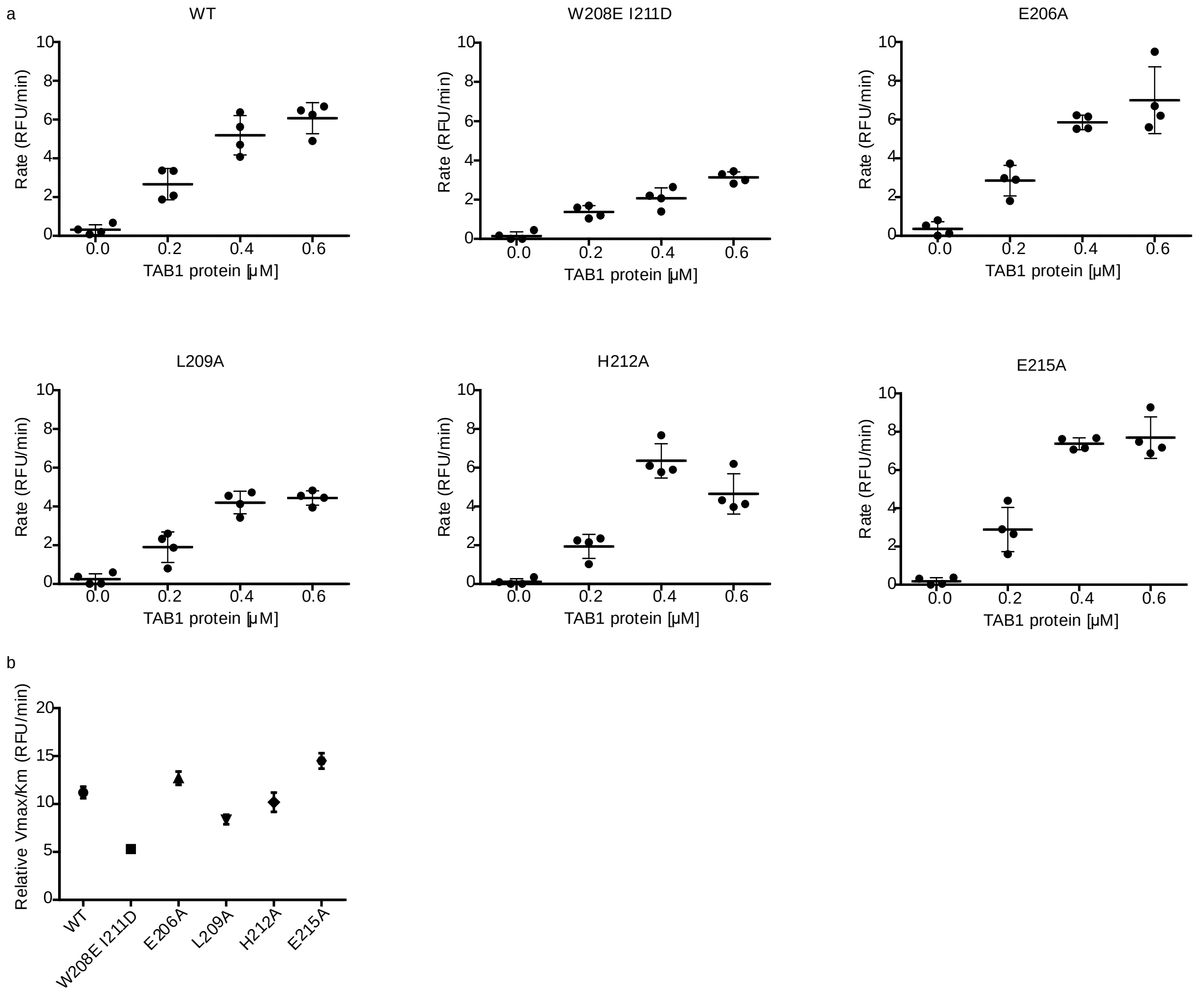

Supplementary Figure 6. Relative second order rate constants of OGT WT and mutants. **(a)** 100 nM OGT wildtype and mutants were incubated in the presence of 0 to 0.6 µM TAB1 protein and 10 µM BODIPY-UDP-GlcNAc. Relative second order rate constants have been generated using a linear regression fit and are noted within the graphs. The error bars represent one standard deviation (SD) from the mean. **(b)** Graph displays the relative second order rate constants of wildtype and mutant OGT displayed in figure **(a)**. The error bars represent +/- errors from the mean. Measurements were performed in quadruplicate and yielded similar data in two biological replicates. Source data are provided as a Source Data file.

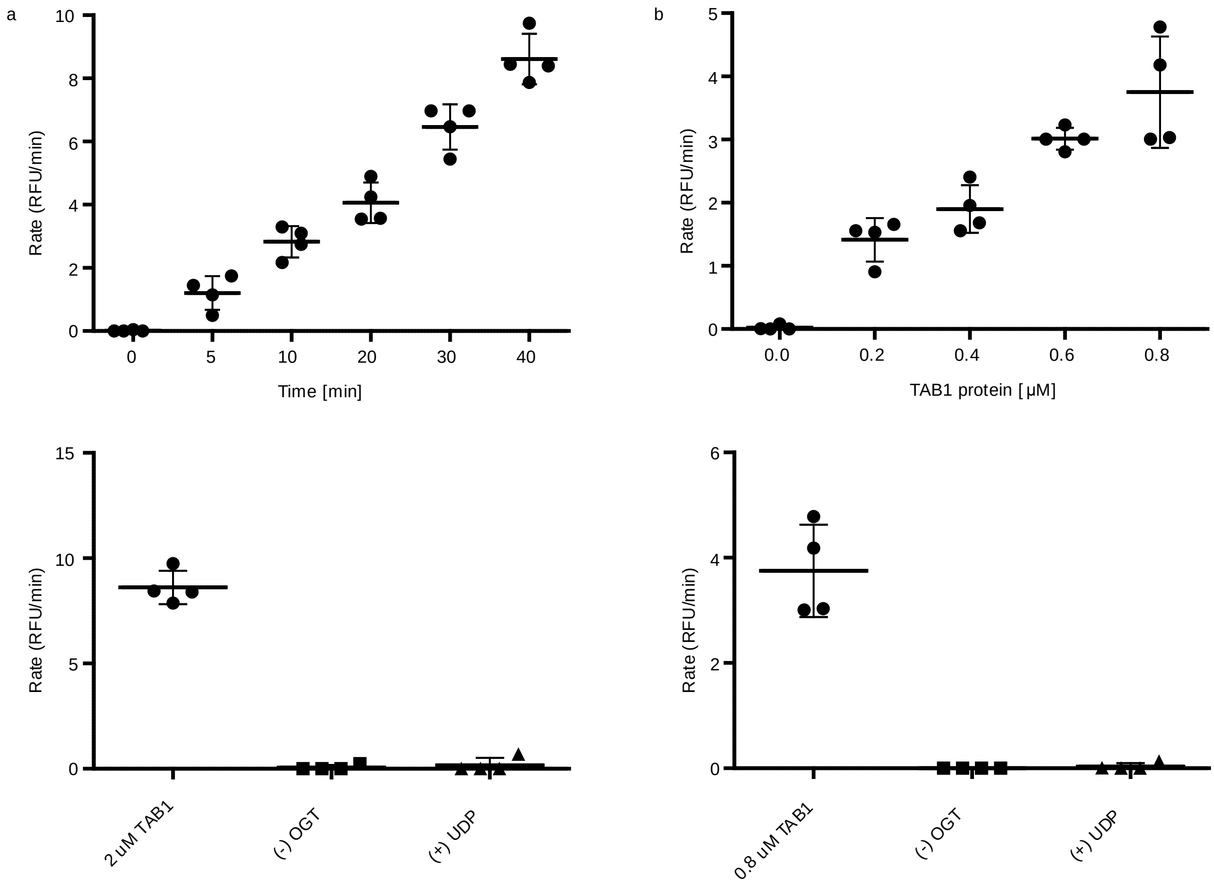

Supplementary Figure 7. Optimizing the OGT activity assay for the use of TAB1 protein. **(a)** A linear rate curve was generated by incubating 100 nM wildtype OGT in the presence of 2 μM TAB1 for 0 to 40 minutes. The below graph compares glycosyl transfer to TAB1 between samples that contain 2 μM TAB1 in the absence of OGT (- OGT) and in the presence of UDP (+UDP) at an incubation period of 40 min. **(b)** A linear rate curve was generated by incubating 100 nM wildtype OGT in the presence of 0 to 0.8 μM TAB1 for 40 min. The below graph compares glycosyl transfer to TAB1 between samples that contain 0.8 μM TAB1 in the absence of OGT and in the presence of UDP at an incubation period of 40 min. Measurements were performed in quadruplicate and yielded similar data in two biological replicates. Error bars one standard deviation (SD) from the mean. Source data are provided as a Source Data file.
