# Supplementary material for: Cryo-EM structure provides insights into the dimer arrangement of the O-linked β-N-acetylglucosamine transferase OGT
Source: Nat Commun. 2021 Nov 11;12:6508. doi: 10.1038/s41467-021-26796-6 (PMC8586251; doi:10.1038/s41467-021-26796-6)
Supplement: Supplementary file 2 — Reporting Summary [file 41467_2021_26796_MOESM2_ESM.pdf]

nature research

Corresponding author(s): Blaza, J.N; Davies, G.J.

Last updated by author(s): Oct 13, 2021

Reporting Summary

Nature Research wishes to improve the reproducibility of the work that we publish. This form provides structure for consistency and transparency in reporting. For further information on Nature Research policies, see our Editorial Policies and the Editorial Policy Checklist.

Statistics

For all statistical analyses, confirm that the following items are present in the figure legend, table legend, main text, or Methods section.

n/a	Confirmed	
☐	☒	The exact sample size (*n*) for each experimental group/condition, given as a discrete number and unit of measurement
☒	☐	A statement on whether measurements were taken from distinct samples or whether the same sample was measured repeatedly
☐	☒	The statistical test(s) used AND whether they are one- or two-sided<br>*Only common tests should be described solely by name; describe more complex techniques in the Methods section.*
☒	☐	A description of all covariates tested
☒	☐	A description of any assumptions or corrections, such as tests of normality and adjustment for multiple comparisons
☐	☒	A full description of the statistical parameters including central tendency (e.g. means) or other basic estimates (e.g. regression coefficient) AND variation (e.g. standard deviation) or associated estimates of uncertainty (e.g. confidence intervals)
☐	☒	For null hypothesis testing, the test statistic (e.g. *F*, *t*, *r*) with confidence intervals, effect sizes, degrees of freedom and *P* value noted<br>*Give P values as exact values whenever suitable.*
☒	☐	For Bayesian analysis, information on the choice of priors and Markov chain Monte Carlo settings
☒	☐	For hierarchical and complex designs, identification of the appropriate level for tests and full reporting of outcomes
☒	☐	Estimates of effect sizes (e.g. Cohen's *d*, Pearson's *r*), indicating how they were calculated

Our web collection on statistics for biologists contains articles on many of the points above.

Software and code

Policy information about availability of computer code

Data collection	EPU (Thermo Fisher Scientific)
Data analysis	MotionCor2, CTFFIND-4.1, RELION 3.0.5, Chimera v.1.14, ChimeraX v.1.2, Coot 0.8.9.2, Flex-EM, Phenix 1.19.1-4122, GraphPad prism 5, Shimadzu LabSolutions 5.85, Astra 7,

For manuscripts utilizing custom algorithms or software that are central to the research but not yet described in published literature, software must be made available to editors and reviewers. We strongly encourage code deposition in a community repository (e.g. GitHub). See the Nature Research guidelines for submitting code & software for further information.

Data

Policy information about availability of data

All manuscripts must include a data availability statement. This statement should provide the following information, where applicable:

- Accession codes, unique identifiers, or web links for publicly available datasets
- A list of figures that have associated raw data
- A description of any restrictions on data availability

Data deposited under PDB ID 7NTF and EMD-12588

Field-specific reporting

Please select the one below that is the best fit for your research. If you are not sure, read the appropriate sections before making your selection.

☒ Life sciences ☐ Behavioural & social sciences ☐ Ecological, evolutionary & environmental sciences

For a reference copy of the document with all sections, see nature.com/documents/nr-reporting-summary-flat.pdf

Life sciences study design

All studies must disclose on these points even when the disclosure is negative.

Sample size	No statistical methods were used to determine sample size for EM data. Sample size was dependent only upon the ability to make adequate 3D reconstructions. For kinetics,
Data exclusions	Removal of poor particles by algorithms and manually curation is part of the data processing pipeline and has been discussed within the paper, notably through careful removal of poorly resolved classes of particles through relion 2D and 3D classifications. Classes were considered poor if reconstructions yielded low resolution features and noise. In doing so, 15,271,670 non-optimal particles were discarded.
Replication	Several different strategies to process the data yielded highly similar 3D reconstructions. 9233 micrographs were collected and processed, however no additional data collections/replicates are presented here for cryo-EM. For kinetics, two biological replicates per experiment yielded similar data.
Randomization	To calculate the gold standard Fourier shell correlation curves, during refinement, the data is randomly split into two halves and refined individually in Relion.
Blinding	Blinding was not relevant to this study since the exact sample under investigation needed to be known.

Reporting for specific materials, systems and methods

We require information from authors about some types of materials, experimental systems and methods used in many studies. Here, indicate whether each material, system or method listed is relevant to your study. If you are not sure if a list item applies to your research, read the appropriate section before selecting a response.

Materials & experimental systems

n/a	Involved in the study	
☒	☐	Antibodies
☒	☐	Eukaryotic cell lines
☒	☐	Palaeontology and archaeology
☒	☐	Animals and other organisms
☒	☐	Human research participants
☒	☐	Clinical data
☒	☐	Dual use research of concern

Methods

n/a	Involved in the study	
☒	☐	ChIP-seq
☒	☐	Flow cytometry
☒	☐	MRI-based neuroimaging
